# Recent Progress in Brillouin Scattering Based Fiber Sensors

**DOI:** 10.3390/s110404152

**Published:** 2011-04-07

**Authors:** Xiaoyi Bao, Liang Chen

**Affiliations:** Physics Department, University of Ottawa, Ottawa, ON K1N6N5, Canada; E-Mail: lchen@uottawa.ca

**Keywords:** fiber optic sensors, Brillouin scattering, polarization mode dispersion, strain, temperature, dynamic measurement, structural health monitoring, birefringence, acoustic wave

## Abstract

Brillouin scattering in optical fiber describes the interaction of an electro-magnetic field (photon) with a characteristic density variation of the fiber. When the electric field amplitude of an optical beam (so-called pump wave), and another wave is introduced at the downshifted Brillouin frequency (namely Stokes wave), the beating between the pump and Stokes waves creates a modified density change via the electrostriction effect, resulting in so-called the stimulated Brillouin scattering. The density variation is associated with a mechanical acoustic wave; and it may be affected by local temperature, strain, and vibration which induce changes in the fiber effective refractive index and sound velocity. Through the measurement of the static or dynamic changes in Brillouin frequency along the fiber one can realize a distributed fiber sensor for local temperature, strain and vibration over tens or hundreds of kilometers. This paper reviews the progress on improving sensing performance parameters like spatial resolution, sensing length limitation and simultaneous temperature and strain measurement. These kinds of sensors can be used in civil structural monitoring of pipelines, bridges, dams, and railroads for disaster prevention. Analogous to the static Bragg grating, one can write a moving Brillouin grating in fibers, with the lifetime of the acoustic wave. The length of the Brillouin grating can be controlled by the writing pulses at any position in fibers. Such gratings can be used to measure changes in birefringence, which is an important parameter in fiber communications. Applications for this kind of sensor can be found in aerospace, material processing and fine structures.

## 1. Introduction

In the past decade, the demand for safe civil infrastructure and power supply has dramatically increased in our society. The pressure on civil engineering, oil and utility industries has grown similarly. Our society requires not only a growth in the supply of services but also improvement in the safety of the supply chain. Disaster prevention requires that engineers design and maintain civil engineering structures based on stress integrity assessments. These requirements turn structural health monitoring (SHM) into a key element of industrial businesses. Clearly there is a need for a technique that allows distributed temperature and strain measurements to be captured in real time over lengths of a few meters to tens of kilometers, even hundreds of kilometers. The distributed sensing technique has the advantage of satisfying all these requirements.

For over two decades, distributed optical fiber sensors based on Brillouin scattering have gained significant interest for their ability to monitor temperature and strain in large infrastructures and replace thousands of point sensors. This kind of sensor has found applications in civil structures, environmental monitoring, the aerospace industry, power generator monitoring and geotechnical engineering. Brillouin scattering occurs as a result of refractive-index fluctuations caused by quasi-coherent acoustic waves initiated from thermally generated sound wave agitations that are capable of scattering incident lightwaves with shifted frequencies. Stimulated Brillouin scattering (SBS) enhances such scattering in a Brillouin optical time domain analysis (BOTDA) [1–8] with intense signal and better spatial resolution when compared with spontaneous scattering based Brillouin optical time domain reflectometry (BOTDR) [9–11]. The highest power of the pump wave is limited by the nonlinear effect in fiber [12,13]. At present, there are two schemes to realize a BOTDA, including Brillouin gain [1–4,6,7] and Brillouin loss [8]. Their working principles will be explained in detail in Section 2.

The performance of distributed sensors based on Brillouin scattering has been improved significantly over the years. The highest spatial resolution over two kilometer sensing length is 2 cm and the longest reported sensing length is 150 km with 2 m spatial resolution and 1 °C temperature resolution, this will be described in Section 12. Simultaneous temperature and strain sensing has been realized in photonics crystal fiber (PCF) and polarization maintaining fiber (PMF) at centimeter spatial resolution.

This review paper is an attempt to summarize the evolution of the distributed sensor based on Brillouin scattering, and the milestones of the distributed sensor in terms of sensing range, spatial resolution, and temperature/strain resolutions, so that readers will have a glimpse of the advantages, limitations and realized potential of this technology. To this end, applications of distributed Brillouin sensors for structural health monitoring have been summarized, and recent research efforts to measure birefringence and its dependence on strain and temperature using Brillouin gratings in polarization maintaining fiber (PMF) have also been included.

The paper is organized as following: The definition of a distributed Brillouin sensor is provided in Section 2, with background information about Brillouin scattering, as well as the temperature and strain dependence of the Brillouin frequency presented in Section 3. Stokes and anti-Stokes wave derivation in optical fiber are included in Section 4. The challenge of long sensing range is to have long sensing length, and high spatial and strain/temperature resolution. After long fiber length induced high fiber loss, low signal to noise ratio (SNR) would create low temperature or strain resolution, as the temperature resolution is proportional to SNR. The high spatial resolution means short Brillouin interaction length which makers the signal even weaker, and sensor performance will be poorer. A few approaches have been proposed to boost the power loss due to the fiber attenuation with Raman amplifiers and they are summarized in Section 5.

Achieving high spatial resolution remains the greatest challenge in designing a distributed sensor. Early efforts have been focused on post-data processing schemes to reduce the spatial resolution to a fraction of the pulse length, *i.e.*, 10–50 cm, those works, based on the same measurement hardware are summarized in Section 6. Following the 1st demonstration of narrow linewidth Brillouin scattering by pre-pumping acoustic wave, different approaches based on the same principle have been proposed leading to recent breakthroughs in the distributed sensor of 2–5 cm resolution over a few kilometers as described in Section 7. As a result, spatial resolution becomes limited by the convolution of the digitizer and pulse generator bandwidths, rather than any physics based mechanism.

To further improve the spatial resolution, an alternative approach utilizing two continuous waves with the difference of the Brillouin frequency counter-propagating in the fiber, and the spatial information is provided by swept the probe wave at various frequency, has been proposed and it allows millimeter spatial resolution with the pulses of 10 ns. This technique is described Section 8.

Section 9 explains the limitation of the temperature and strain resolution and the fiber birefringence effect on temperature and strain resolution.

Because the Brillouin frequency is a function of temperature and strain, often the temperature measurements assume that strain is constant; while for strain measurement; temperature is treated as a constant. Such an assumption may not be applicable during field tests, hence different approaches of simultaneous temperature and strain measurement have been discussed in Section 10 using the power, linewidth and multi-Brillouin peak features in addition to the Brillouin peak frequency.

Sections 11 and 12 summarize two novel approaches to achieve high performance distributed sensor systems based on sensing length, spatial resolution and temperature/strain resolution over 100 km sensing length without an inline amplifier. Section 13 gives a unique approach for combing the Brillouin gain and loss for distributed sensor, and it establishes a model for four-wave-mixing in fiber via SBS, which serves as foundation for Section 14 on the applications of Brillouin gratings in optical fiber. Section 15 is on dynamic strain measurement with BOTDA, and Section 16 summarizes the effort of the structural health monitoring with Brillouin scattering based distributed sensor. The final section, Section 17, gives the remarks and prospect of future research subjects.

## 2. Distributed Fiber Sensors Based on Brillouin Scattering

This section explains the concept of distributed sensors based on Brillouin scattering and the best performance of a Brillouin scattering based distributed sensor under the limitation of the acoustic wave relaxation time.

A distributed fiber sensor based on Brillouin scattering exploits the interaction of light with acoustic phonons propagating in the fiber core. The Brillouin scattered light has a frequency shift proportional to the local velocity of the acoustic phonons (also called acoustic waves), which depends on the local density and tension of the glass and thus on the material temperature and strain. This Brillouin frequency shift is on the order of 9–13 GHz for radiation wavelengths of 1.3–1.6 micro-meters in standard single mode communication fibers, and it is given approximately by:

(1) $$\upsilon_{B}\;(z)=\frac{{2n}_{\mathrm{eff}}\;(z)V_{a}}{\lambda }$$

where *n_eff_* is the effective mode refractive index of the fiber as a function of distance z. The velocity of sound in glass is *V_a_* and *λ* is the free-space wavelength. The sensing capability of this scattering phenomenon arises from measurement of the distributed Brillouin frequency shift dependence on both strain and temperature.

The concept of using Brillouin scattering in fiber for optical sensing was first proposed in 1989 [1] and it was termed Brillouin optical time domain analysis (BOTDA), using the pump-probe wave approach. This basic approach involved launching a short pump pulse into one end of the test fiber and a CW (continuous wave) probe beam into the other end. If the probe wave is at the Stokes frequency, then energy flows from the pump to the Stokes wave providing Brillouin gain to this CW wave. If the probe wave instead takes the anti-Stokes frequency, it then gives energy to the pump wave (pulsed signal), and the detected CW signal experiences a Brillouin loss.

The frequency difference between the two lasers could be set to a particular value corresponding to the Brillouin frequency of the optical fibers, and the CW probe would experience gain varying along the fiber. The gain as a function of position along the fiber could thus be determined by the time dependence of the detected CW light. By measuring the time dependent CW signal over a wide range of frequency differences between the pump and probe, the Brillouin frequency at each fiber location could be determined. This allowed mapping the strain or temperature distribution along the entire fiber length.

The first strain distribution measurement was reported on submarine cables [2] using distributed fiber sensors based on Brillouin scattering. The reported strain distribution was obtained over a 1.3-km cable [3]. Later, another strain measurement on the bent slot-type optical cables was reported [4]. Temperature measurement using Brillouin scattering was proposed in 1989 [5] utilizing the linear relationship between Brillouin frequency shift and temperature in a single mode fiber and measured with a Fabry-Perot interferometer. Then, a distributed temperature measurement on a 1.2 km single mode fiber with a 3 °C temperature resolution and a 100 m spatial resolution was demonstrated with a BOTDA system [6]. In the next three years this performance had improved to achieve a 22 km sensing length with 5 m spatial resolution of 1 °C temperature resolution [7].

The next development in BOTDA was the use of Brillouin loss rather than Brillouin gain [8] in order to increase the sensing length. Relative to the pulsed pump wave, the CW probe wave has been set at the anti-Stokes frequency instead of the Stokes frequency; hence the detected signal is a Brillouin loss. The difference between the gain and loss is the pulse signal has been switched from “donor” to “receiver” with respect to energy exchange. For long sensing lengths (>10 km), there are two limitations of the former method: (1) peak pulse power of the pump must be lower than the stimulated Brillouin scattering (SBS) threshold; and (2) the finite energy of pump pulses can become significantly depleted, leading to uneven gain along the sensing fiber excessive depletion occurs at the beginning.

Under the Brillouin loss regime, the CW probe therefore experienced loss at locations along the fiber at which the frequency difference between the lasers matched the local Brillouin frequency of the fiber. As it is much harder to deplete the CW rather than a pulsed pump, a longer sensing length of 50 km with 5 m spatial resolution and 1 °C temperature resolution was reported using the Brillouin loss mechanism [8]. The longest reported distributed sensor length using BOTDR is 57 km with a spatial resolution of 20 m and 3 °C temperature resolution [9]. More recently, a sensing length of 150 km was demonstrated by amplifying the BOTDR signal using Raman amplifiers in the fiber [10]; a temperature resolution of 5.2 °C was achieved with a 50 m spatial resolution. The better spatial resolution in BOTDA is attributed by the pump and probe wave interaction induced Brillouin amplification over the entire sensing length, while in BOTDR only one pump pulse is used and it works in spontaneous Brillouin scattering regime.

## 3. Spontaneous and Stimulated Brillouin Scattering (BS) in Single Mode Optical Fibers

This section provides mathematical equations for the spontaneous and stimulated Brillouin scattering, to illustrate the temperature and strain dependence of the Brillouin frequency, as well as the amplification of the pump or probe wave via Brillouin gain or Brillouin loss resulting from SBS induced by electrostriction.

Brillouin scattering is caused by the collective acoustic oscillations of the solid state matter. From the microscopic point of view, the intermolecular interaction in the solid state matter creates a tendency for molecules to stay at some stable separation distance from each other. There is an energy penalty when the actual intermolecular distance is either farther apart or closer than this stable separation. This microscopic existence of balanced intermolecular distances would set a new collective motion. Imagine if a neighboring molecule becomes closer than stability allows, then it will be pushed away towards the point of stability. However, when it reaches that position it will not stop, rather it will overshoot passing the stable separation distance. Once it is farther away it will experience an attraction to pull it back toward the optimum position. However it will again overshoot when it returns. Such a repeating cycle forms a collective motion called acoustic phonons. To describe the above process, we need to use macroscopic parameters, such as the density, pressure and temperature of the matter. As the acoustic wave induces local pressure changes, the local density *ρ* is affected, altering the material’s polarizability and allowing for direct macroscopic characterization via the Maxwell equations.

### 3.1. Spontaneous BS

The acoustic wave is captured in the following wave equation [14] describing the pressure wave with the local pressure variation parameter Δ*p̃*:

(2) $$\frac{\partial^{2}\Delta \tilde{P}}{{\partial t}^{2}}-\Gamma \prime \nabla^{2}\frac{\partial \Delta \tilde{P}}{\partial t}-V_{a}^{2}\nabla^{2}\Delta \tilde{P}=0$$

Here Γ′ is a damping parameter related the local viscosity of the material, while *V_a_* is the sound velocity. To better understand the nature of an acoustic wave, we can find a simple solution for a one-dimensional propagating wave:

(3) $$\Delta \tilde{P}=\Delta {\mathrm{Pe}}^{i(\mathrm{qz}-\Omega t)}+c.c.$$

Here, *c.c.* stands for complex conjugate. We obtain the following relation governing the propagation constant *q* and angular frequency Ω,

(4) $$-\Omega^{2}-i\Gamma \prime q^{2}\Omega +V_{a}q^{2}=0$$

This gives the solution:

(5) $$q^{2}=\frac{\Omega^{2}}{V_{a}^{2}-i\Gamma \prime \Omega }\approx {\left(\frac{\Omega }{V_{a}}\right)}^{2}\left(1+i\frac{\Omega \Gamma \prime }{V_{a}^{2}}\right)$$

It illustrates that the acoustic wave has a complex propagation constant q describing the usual attenuation expected from our physical intuition. From the energy conservation prospective, this kind of attenuation reflects nothing but the energy transferring processes. The acoustic wave is necessarily coupled to other types of modes of the system. At the level of the thermodynamic noise, all the modes have non-zero intensity in a system. This type of noise fluctuation serves as the seed for the spontaneous Brillouin scattering process. In order to describe this process more quantitatively, we assume a polarizability change associated with an acoustic wave in the following form:

(6) $$\Delta ɛ(r,t)=\Delta \bar{ɛ}e^{i(qr-\Omega t)}+c.c.$$

Also, if we assume that we have a plane EM wave of the form:

(7) $$E(r,t)=E_{0}e^{i(kr-\omega t)}+c.c.$$

Then one can find the corresponding polarization change:

(8) $$\Delta P(r,t)=\Delta ɛ(r,t)E(r,t)=\Delta \bar{ɛ}E_{0}e^{i\left[(k+q)r-(\omega +\Omega )t\right]}+c.c.+\Delta \bar{ɛ}E_{0}e^{i\left[(k-q)r-(\omega -\Omega )t\right]}+c.c.$$

Because of the interaction between the acoustic wave and the incident light, the anti-Stokes (first term in the above expression) and Stokes (second term in the above expression) waves are *naturally created.* For the anti-Stokes wave, we have the following momentum and angular frequency relationships:

(9) $$\begin{array}{l} k\prime =k+q\\ \omega \prime =\omega +\Omega \end{array}$$

And for the Stokes wave, we have correspondingly:

(10) $$\begin{array}{l} k\prime =k-q\\ \omega \prime =\omega -\Omega \end{array}$$

We recall that the dispersion relationship between the propagation constant and angular frequency of a light wave is quantified by the refractive index:

(11) $$\begin{array}{cccc} k=n(\omega )\frac{\omega }{c}, & & & k\prime =n(\omega \prime )\frac{\omega \prime }{c}, \end{array}$$

while the dispersion for acoustic waves is described by $q=\frac{\Omega }{V_{a}}$.

Acoustic angular frequencies are much smaller than optical frequencies, Ω ≪ ω, Ω′ ≪ ω, where *k*′ and *k* propagate in opposite directions along the fiber. Considering the case of anti-Stokes Brillouin scattering, and assuming acoustic wave propagation in the +z direction, we have the following momentum conservation for the anti-Stokes domain:

(12) $$\begin{array}{l} n(\omega \prime )\frac{\omega \prime }{c}=-n(\omega )\frac{\omega }{c}+\frac{\Omega }{V_{a}}=n(\omega +\Omega )\frac{\omega +\Omega }{c}\\ ≅n(\omega )\frac{\omega }{c}+\frac{\Omega }{c}\frac{d[n(\omega )\omega ]}{d\omega }≅n(\omega )\frac{\omega }{c}+\frac{\Omega }{c}n_{g}(\omega ) \end{array}$$

Here, *n_g_* (*ω*) is the group refractive index. Let us solve for the Brillouin angular frequency:

(13) $$\begin{array}{l} \Omega ≅\frac{2n(\omega )\omega }{\frac{c}{V_{a}}-n_{g}\left(\omega \right)}\approx 2n(\omega )\frac{V_{a}}{c}+2n(\omega )n_{g}(\omega )\omega {\left(\frac{V_{a}}{c}\right)}^{2}\\ =2\pi \frac{2n(\omega )V_{a}}{\lambda }+2\pi \frac{2n(\omega )V_{a}}{\lambda }n_{g}(\omega )\frac{V_{a}}{c} \end{array}$$

We see that to the first order of $\frac{V_{a}}{c}$, the Brillouin angular frequency in the anti-Stokes domain is proportional to the acoustic velocity *V_a_* and the local phase refractive index, while being inversely proportional to wavelength *λ*.

However, to the second order ${\left(\frac{V_{a}}{c}\right)}^{2}$ we find an extra term, and this term is of different sign in the Stokes domain. Therefore, the Brillouin angular frequency shift in the anti-Stokes domain is different from that in the Stokes domain. This difference can be verified experimentally and is useful in measuring chromatic dispersion, and could be potentially for measurement of polarization mode dispersion through group velocity measurement, because the Brillouin gain is proportional to local state of polarization (SOP) of the pump and probe wave.

Fiber sensors based on Brillouin scattering (mostly ignore the second order effect) utilize the fact that the Brillouin frequency is proportional to the material's local thermodynamic properties such as sound velocity and phase refractive index, two quantities which in turn depend on local temperature and strain. Over quite a large range of temperature and strain, the Brillouin frequency shifts of most fibers are proportional to local changes in temperature *ΔT* and strain *Δɛ*, *i.e.*, ΔΩ = *C_T_*Δ*T* + *C_ɛ_*Δ*ɛ*. Here, *C_T_* is the temperature coefficient and *C_ɛ_* is the strain coefficient for the fiber. Thus, variations in either temperature or strain can cause the Brillouin frequency to change, hence making it difficult to distinguish between these two effects, if one measures the Brillouin frequency change alone. To overcome this problem, various methods have been proposed to measure temperature and strain simultaneously using specialty fibers. This will be explained in Section 10.

### 3.2. Stimulated BS and Electrostriction

Electrostrictive pressure results from the propagation of two lightwaves in a fiber medium. The frequency difference between two optical waves equals the induced acoustic wave frequency, namely, the Brillouin frequency. The incident and Stokes waves produce a beat signal at the Brillouin frequency, which then induces a density wave enhancing the acoustic wave and consequently increasing the number of phonons. This provides an improved efficiency of the scattering process characterized as operating in the stimulated regime. With a high power of single pump wave, SBS can be created when the pump power is above the threshold value, and it is initiated from spontaneous scattering, the medium is said to be a Brillouin generator. SBS can also be generated by launching another beam from the opposite end of the material at the Stokes frequency, in addition to the pump wave. This is the so-called Stokes signal, while the input is called the pump signal. The Maxwell equations can be used to describe the beat signal induced nonlinear polarization contribution via the electrostriction effect that is associated with the mass density change *Δρ* as follows:

(14) $$P_{\mathrm{NL}}={ɛ}_{0}\Delta \in E={ɛ}_{0}\varrho_{0}\left(\frac{\Delta \in }{\Delta \varrho }\right)\frac{\Delta \varrho }{\varrho_{0}}E=\frac{{ɛ}_{0}\gamma_{e}}{\varrho_{0}}\Delta \varrho E$$

(15) $$\frac{\partial^{2}E}{{\partial z}^{2}}-\frac{1}{{\left(c/n\right)}^{2}}\frac{\partial^{2}E}{{\partial t}^{2}}=\frac{\gamma_{e}}{c^{2}\varrho_{0}}\frac{\partial^{2}\left[\Delta \varrho E\right]}{\partial t^{2}}$$

Here *ϱ*_0_ denotes the mean mass density of the material, and Δ*ϱ* is the mass density variation associated with the acoustic waves. The equation governing the acoustic waves is directly quoted from the book by reference [14]. The above equations form the foundation for the gain and loss formulas presented in Section 14 for the transient four wave mixing process of SBS and in Section 15 for the static condition of Brillouin gratings in optical fiber.

## 4. Stokes and Anti-Stokes Frequency Shift

From the preceding derivation of the density fluctuation, we see that temperature and strain dependence arise from pressure and density waves, which are also associated with birefringence of the optical fiber via *n_eff_* and sound velocity. Both of them change with temperature and strain. Because the birefringence dependence varies with frequency, the dependence of the temperature and strain could be different for Stokes and anti-Stokes waves, as they are associated with two different gratings representing opposite traveling directions of the acoustic wave. Note such a change depends on fiber position, which could lead to a position dependent temperature and strain coefficients, namely, *C_T_(z)*, and *C_ɛ_ (z)*. The introduction of the polarization scrambler may reduce such position dependence, and increase the accuracy for the temperature and strain measurement.

The Stokes and anti-Stokes Brillouin frequency can also be derived based on the relativistic Doppler effect, and its first order expansion gives the same result as momentum conservation of the Stokes and anti-Stokes frequencies for Brillouin scattering. Because of the slight frequency difference between the anti-Stokes and Stokes domains, it is very difficult to measure. The common measurement makes use of two processes: Brillouin gain for the anti-Stokes frequency and Brillouin loss for the Stokes frequency. Note that during the two processes the optical frequency of the probe wave in the Brillouin gain must be kept the same as that of the pump wave in the Brillouin loss process, so that the Stokes and anti-Stokes domain of the Brillouin frequencies are relative to the same carrier [15,16].

**Figure 1.**
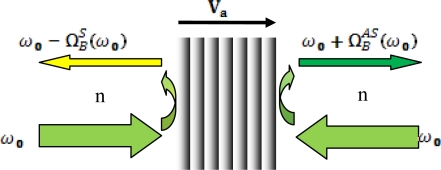
Schematic diagram for the Doppler effect.

Brillouin scattering viewed on the basis of the relativistic Doppler effect can be illustrated as in Figure 1.

As plotted we describe the Brillouin scattering in a view that light of angular frequency *ω*_0_ is incident on a moving acoustic wave at speed *V_a_*, and then a head-on collision creates reflected light (anti-Stokes) with an angular frequency shifted upward according to the relativistic Doppler effect, to be precise, we include the dispersion in refractive index *n* for optical fibers, as the single mode fiber has non-negligible dispersion which can be account through n:

(16) $$\omega_{0}+\Omega_{B}^{\mathrm{AS}}(\omega_{0})=\omega_{0}\sqrt{\frac{1+\frac{n(\omega_{0})V_{a}}{c}}{1-\frac{n(\omega_{0})V_{a}}{c}}}\sqrt{\frac{1+\frac{n[\omega_{0}+\Omega_{B}^{\mathrm{AS}}(\omega_{0})]V_{a}}{c}}{1-\frac{n[\omega_{0}+\Omega_{B}^{\mathrm{AS}}(\omega_{0})]V_{a}}{c}}}$$

Here, *n(ω_0_)* is the refractive index of the fiber at frequency ω_0_, and *c* is the speed of light in vacuum. Similarly, the tail-end collision induces a reflected light (Stokes) with an angular frequency shifted downward by:

(17) $$\omega_{0}-\Omega_{B}^{S}(\omega_{0})=\omega_{0}\sqrt{\frac{1-\frac{n(\omega_{0})V_{a}}{c}}{1+\frac{n(\omega_{0})V_{a}}{c}}}\sqrt{\frac{1-\frac{n[\omega_{0}-\Omega_{B}^{S}(\omega_{0})]V_{a}}{c}}{1+\frac{n[\omega_{0}-\Omega_{B}^{S}(\omega_{0})]V_{a}}{c}}}$$

Obviously the Stokes angular frequency $\Omega_{B}^{S}(\omega_{0})$ and anti-Stokes angular frequency $\Omega_{B}^{\mathrm{AS}}(\omega_{0})$ must be different according to the solutions of Equations (16) and (17). The second view of unequal Stokes and anti-Stokes domain Brillouin frequency shift is the conventional energy and momentum conservations that are widely used in the discussion of spontaneous and stimulated Brillouin scattering as illustrated in the previous section. The frequency difference between Stokes and anti-Stokes wave, ${\Delta \Omega }_{B}=\Omega_{B}^{\mathrm{AS}}-\Omega_{B}^{S}$, is measured in single mode fiber (SMF) in both stimulated Brillouin scattering (SBS) and spontaneous BS at a wavelength of 1,550 nm. Experimental results by our group show that in SBS this value changes with varied state of polarization (SOP) and polarization scrambling of the pump light, the minimum and maximum of ΔΩ*_B_* is 0.490 MHz and 0.715 MHz; while in spontaneous BS this value varies between 0.516 MHz and 0.555 MHz for an SNR of 60 dB.

## 5. Long Sensing Length for the Distributed Fiber Sensors with BOTDA

**Figure 2.**
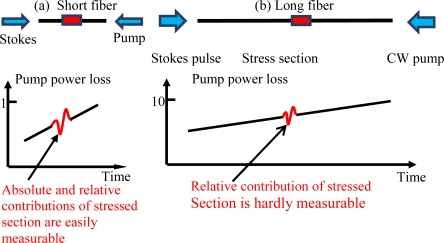
Small stress response in short and long fiber lengths.

Having established the theoretical background of BOTDR and BOTDA, in this section we will explain the evaluation of the distributed sensor system. In general, high optical power is required for both pump and probe waves to overcome the fiber loss; this however may introduce uneven Brillouin gain along the optical fiber. As a uniform signal to noise ratio (SNR) is required from the design perspective of a distributed sensor, low gain across a long sensing length is needed. This can be illustrated in Figure 2. One compromise of the low gain system over the long sensing length is that there is a correspondingly low SNR associated with small sections of fiber, compared with that of short sensing lengths. Hence the spatial resolution or temperature (strain) resolution are compromised for long sensing length.

In order to maintain low Brillouin gain with high SNR, the pump power should be kept at a minimum and probe power maximized, but kept below the onset of modulation instability (MI) [13]. MI is a process that the amplitude and phase modulation of the wave grow as a result of interplay between nonlinearity and anomalous dispersion. In the frequency domain MI leads to the generation of sidebands symmetrically placed about the pump frequency, hence the energy in the high power pump or probe waves will be transferred to those sidebands instead of contributing to the Brillouin gain or loss process [11,12]. It is also known that via MI new wavelengths can be generated in conventional optical fibers by parametric four-wave mixing (FWM) when they are pumped close to the zero-dispersion wavelength, as the phase-matching condition is sensitive to the exact shape of the dispersion curve, and it can be obtained only in the normal dispersion regime when nonlinear effects are neglected. To avoid the MI effect, a dispersion shifted fiber with normal dispersion has been used [17] in long sensing range.

An alternative solution is to use an optical pulse coding technique to enhance the SNR [16,18]. For example, 2 MHz Brillouin frequency resolution, and 1 m spatial resolution were achieved using non-return-to-zero (NRZ) coded pulses over 50 km of SMF-28 fiber [20], while 0.5 m spatial resolution combined with 0.7 °C temperature resolution were obtained using return-to-zero (RZ) code over a 50 km LEAF (Large Effective Area Fiber of Corning Inc.) fiber [21]. Because the RZ coded pulses have minimized nonlinear effects, better spatial resolution and Brillouin frequency resolution are achieved. Also, since there are 4 Brillouin peaks in LEAF fiber, higher input pump power can be launched, thereby yielding better spatial and temperature resolution, with sub-meter spatial resolution using RZ coded pulses.

For fiber lengths beyond 60 km, an inline amplifier has been applied for a BOTDA based sensor system. Recently, inline Raman amplifiers were used to extend the sensing length to 75 km obtaining spatial resolutions of 2 m of 75 km [22] and 2 m of 120 km with bi-direction of amplification [23] 10 m over 75 km [24], as well as 13 m of 46 km sensing length [25] for hybrid FBG points and distributed sensor, respectively. Pump depletion could be avoided by low pump and probe power levels with the fiber loss being compensated by the distributed Raman gain. Although the Raman amplification has allowed for longer sensing lengths, the noise introduced by 2nd order Rayleigh scattering of Raman amplification has the same frequency as that of the Brillouin pump or probe waves depending on uni- or bi-directional Raman amplification. As a result, spatial and temperature resolution have been significantly compromised due to the low SNR in above demonstrations.

By dividing the entire sensing length into several sensing sections and then recovering them through frequency division multiplexing (FDM) [26] of BOTDA or time division multiplexing (TDM) [27], one can obtain similar or even better performance of the distributed sensor without Raman assisted BOTDA. This means the Brillouin interaction happens only within the sensing section having maximum Brillouin gain, while the remaining sections are only subjected to the fiber loss. In FDM, fibers with different Brillouin frequency shifts at distinct sections reduce the effective Brillouin amplification length to one resonant Brillouin frequency section rather than the entire sensing fiber. Thus, a moderate pump wave can be used to enhance the Brillouin interaction in the individual section without pump depletion or excessive amplification of the probe wave, due to the short Brillouin interaction length for a specific section. Therefore, the limitation of self-phase-modulation (SPM) effect is also mitigated due to the “short interaction length” for a specific Brillouin frequency section or a specific long pump pulse covered section length,

For the detection of the CW, the low power should be used, so that highest contrast can be obtained with high power of the pulse wave. Because of the relatively short effective sensing length, the true Brillouin gain in the section is in fact larger than the gain if it was operated for the entire sensing length. Multiplexing is realized by sweeping a larger Brillouin frequency range which spans different locations. Because of larger Brillouin gain in each individual section, SNR has been improved in comparison with long sensing length using coded pulse schemes and Raman amplification assisted long distance BOTDA. Based on this concept we demonstrated a 75 km BOTDA using three types of 25 km fiber with a spatial resolution of 1 m and an accuracy of 1 °C/20 μɛ at the end of 75 km, and a spatial resolution of 0.5 m and an accuracy of 0.7 °C/14 μɛ at the end of 50 km [25]. The limitation of this technique is the long measuring time required due to the larger Brillouin frequency range.

In TDM, the restriction of the effective length is realized by two pulses: both pump and probe pulses, in which the pump pulse provides the effective length for the Brillouin interaction and amplification, while the probe pulse provides the spatial resolution. Using this idea, one can multiplex different sections by delaying the pump pulse time relative to the probe pulse and then the temperature and strain can be mapped. Using this technique, spatial resolutions of 0.6 m and 2 m have been demonstrated at the end of 75 km and 100 km respectively with a Brillouin frequency shift resolution of 2 MHz, which is equivalent to 2 °C temperature resolution.

The high performance of FDM and TDM without inline amplification is due to the fact that pump depletion and gain saturation induced Brillouin gain distortion on the probe wave, are accumulated only within the single section of the time or frequency domain, rather than continuously accumulating over the entire sensing length. This idea is similar to regenerating a communication system's signal, when its SNR no longer meets the accepted level.

## 6. Spatial Resolution Limitation and Improvement by Various Signal Processing Schemes

Spatial resolution is determined by pulse width, and can be improved by using a short pulse; meanwhile, a shorter pulse provides a broadened Brillouin gain spectrum (BGS) and a weaker Brillouin signal, especially, when it is much shorter than the material's phonon lifetime (∼10 ns in silica fiber, which is equivalent to 30 MHz bandwidth of the Brillouin spectrum). As the measured Brillouin spectrum is a convolution of the pulse and the actual Brillouin spectra, for a 1 ns pulse the equivalent pulse spectrum width is 1 GHz. The pump power spreads over 1 GHz instead of 30 MHz resulting in an effective power drop by a factor of 30. Obviously, the SNR will be reduced significantly. Even if high pump power is used to compensate for this power loss and enhance spatial resolution, the measurement accuracy of the Brillouin frequency shift remains low due to difficulty of measuring 1 MHz over such a broadband frequency base. These limitations prohibit high precision distributed sensor operation by simply shortening the pulse-width [28]. Generally, it is regarded that the spatial resolution cannot be better than 1 m for conventional Brillouin time-domain sensors, either BOTDR or BOTDA.

Early efforts to improve spatial resolution have focused on various signal processing schemes. Three such methods are introduced to obtain spatial resolution values shorter than the pulse length by (1) identifying the stress boundary using the compound spectral method; (2) exploiting the second order derivative of frequency and location; and (3) introducing the Rayleigh Equivalent Criterion (REC).

### 6.1. De-Convolution of Compound Brillouin Spectrum to Improve the Spatial Resolution

For a fixed pulse length, one can send pulses twice at different times with one pulse length delay for the second pulse, so that the Brillouin spectrum is de-convoluted in the same position twice to cover the half of the pulse length, which means the spatial resolution is improved by a factor of two [29]. Similar idea can be used to get a factor of four improvements with half of the pulse length delay, so that in any given position, the Brillouin spectrum will be de-convoluted four times. Under uniform strain within the pulse length, the Brillouin loss spectrum is a single peak at a beat frequency corresponding to the strain and temperature in the fiber. The line width depends on the optical pulse length used. If the strain or temperature within the pulse width is non-uniform, the Brillouin loss profile contains multiple peaks. The individual components of the compound spectrum may be resolved using signal processing, if the first section of the strain or temperature is known.

**Figure 3.**
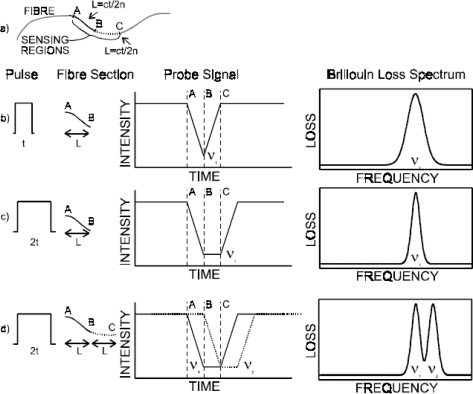
Time-domain waveforms and Brillouin loss spectra for various pulse widths [29] (Copyright © 1999 IEEE, Reprinted with permission).

Assuming the Brillouin gain coefficient is constant over the length of a fiber section under constant strain, the time-domain signal is a convolution of the shape of the section of uniform strain (rectangular) with the shape of the pulse (also approximately rectangular). Considering a fiber section shown in Figure 3a, as the pulse enters the section the signal increases to the point where the pulse is entirely within the section. Then the signal remains at this level for as long as the pulse is within the section, decreasing as the pulse leaves. For a pulse of the same width as the strained section, this results in a triangular waveform (Figure 3b). A pulse twice the width of the section will produce a trapezoidal shape in the time-domain waveform (Figure 3c). If there are two adjacent sections of different uniform strain (and thus different Brillouin frequency), the trapezoidal shape from the latter section will be partially overlapped by that of the earlier section. If the time-domain signal is measured at a point in this overlap, the frequency domain signal will contain the spectra of both sections (Figure 3d). A pulse width twice the length of a single fiber section generates a spectrum containing two Brillouin lines corresponding to the strains of the two sections covered by the pulse. It is possible to use the compound spectra of N sections measured twice at a time, together with a prior knowledge of the signal from one of the sections to determine the Brillouin loss spectrum of the other N-1 sections.

### 6.2. Second-Order Derivative of the Frequency and Position to Identify Strain Change

**Figure 4.**
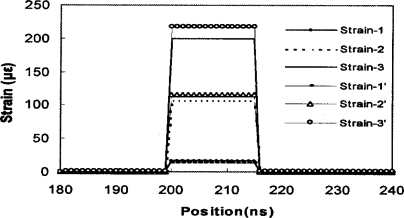
Brillouin loss at three different strains for 2 ns pulse (equivalent to 20 cm spatial resolution [30] (Copyright © 2005 OSA, Reprinted with permission).

Finding the boundary of two strained sections is a difficult task, especially for a large strain gradient or a small strain change. It involves multiple peaks (for large Brillouin frequency variation) or a broadened Brillouin profile (for small Brillouin frequency variation). Between different strains in two sections, there is a point of discontinuity in the Brillouin gain spectrum of the two peaks. As a result, the first derivative of the frequency will pick up this transition point. One can take $\alpha (\upsilon ,z)=\frac{\Delta P}{\Delta \upsilon }=\frac{P(\upsilon +\Delta \upsilon_{\mathrm{scan}},z)-P(\upsilon ,z)}{\Delta \upsilon_{\mathrm{sacn}}}$ [30] to get the first order derivative, where *P(v*,*z)* is the Stokes intensity near the peak frequency and *P*(*υ* + Δ*υ_scan_*, *z*) is the intensity at *υ* + Δ*υ_scan_*, Δ*υ_scan_* is the laser frequency scanning step size. Then calculate the 2^nd^ order partial derivative of the Stokes intensity with respect to position, $\beta (\upsilon ,z)=\frac{\Delta \alpha (\upsilon ,z)}{\Delta z}=\frac{\alpha (\upsilon ,z+\Delta z)-\alpha (\upsilon ,z)}{\Delta z}$, where Δ*z* is the step size of the digitizer. Figure 4 shows data of 20 cm spatial resolution, improved to 5 cm location accuracy with such processing. It is limited by the readout resolution, *i.e.*, the time resolution of the digitizer. This means, the location accuracy is a quarter of the pulse length [30]. Hence one can get four times improvement for the location accuracy without using narrower pulses, which could otherwise require expensive broadband electronics and lead to higher noise. Instead, the signal processing is done by computer programming rather than by hardware.

### 6.3. Rayleigh Equivalent Criteria to Identify a Strain Section Shorter than the Spatial Resolution

**Figure 5.**
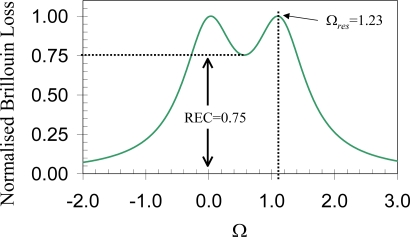
Definition of the REC for simulated Brillouin loss spectrum with the fiber length of 1,000 m, z = 0, Pump = 30 mW, Probe = 5 mW, pulse length = 20 m [31] (Copyright © 2005 IEEE, Reprinted with permission).

The optical imaging Rayleigh criterion allows for determination of the smallest resolvable angular separation of two identical objects. It uses two distributions of equal intensity, whose equations have the generic form *I* = sinc^2^ *δ*, where the phase *δ* is related to the distance between two objects. This criterion assumes that these two peaks can be resolved as soon as the maximum intensity of the first peak coincides with the first minimum of the second peak, which happens at *δ* = π. The separation between these two objects is then defined as the smallest resolvable distance. The intensity at this minimum distance is 8/π^2^. In a similar fashion, this idea can be applied to the distributed Brillouin sensor in the Brillouin gain or loss spectrum to identify the two separated strain or temperature peaks. The original intensity is replaced by the Brillouin spectrum, while the phase is replaced by the Brillouin frequency. Figure 5 shows the Brillouin loss spectrum obtained by simulation of the phenomenological model [31]. The dip amplitude (minimum of the Brillouin loss spectrum between the two peaks) is 0.75 corresponding to Ω*_Bs_* = Ω*_res_* = 1.13. Here Ω*_Bs_* = (*ν_Bs_* − *ν_B_*)/Δ*ν_B_*, and Ω*_res_* represents the smallest resolvable frequency difference. Δ*ν_B_* is the bandwidth of Brillouin spectrum; *ν_Bs_* is the Brillouin frequency of the resonance, and *ν_B_* is the Brillouin spectrum’s central frequency. The dip amplitude is defined as the Rayleigh Equivalent Criterion (REC).

**Figure 6.**
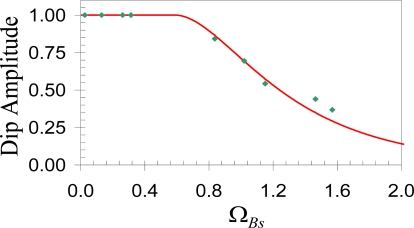
Normalized Brillouin loss spectrum dip plotted as a function of the normalized Brillouin frequency shift. Simulation results (plain curve) and experimental data (diamond) at the middle of sensing length 40 m, Ppump = 5 mW, Pcw = 3 mW, pulse length of 20 cm, ER = 11 dB [31] (Copyright © 2005 IEEE, Reprinted with permission).

Figure 6 represents experimental data obtained within controlled laboratory conditions. A section of 1.5 m out of 40 m is subjected to traction by applying a linearly increasing weight. The unstrained fiber’s Brillouin frequency is 12,819.98 MHz. By introducing the REC, the minimum spatially resolvable stress section is reduced to ½ the spatial resolution, with the Brillouin frequency uncertainty of 5% compared to the normal spatial resolution, which equals to the pulse width. Apparently, the REC is an efficient threshold to unambiguously detect stress sections that are shorter than spatial resolution with an uncertainty lower than 5%. Thus using computer processing to apply the REC without introducing hardware changes can improve the location accuracy by a factor of two.

## 7. Spatial Resolution Improvement by Pre-Pumping the Acoustic Wave

In recent years, great attention has been paid to improving spatial resolution, and several novel techniques have been proposed, bringing spatial resolution to the order of cm [33–37]. Among these techniques, there is one common working principle of pre-excitation, which was first observed in [33]. In this method, a pre-activated acoustic field is created via electrostriction through the interference of a CW pump wave (or a long duration pulse), and a counter-propagating CW signal wave, having a locally matched Brillouin frequency shift with respect to the pump wave. When the acoustic wave is developed with a natural Brillouin line-width, a very short pulse (ns) arrives and takes a sample of the electrostriction effect created acoustic wave in the narrow line-width. In this way, the Stokes signal is much stronger than its amplified portion, generated by the short pulse interaction with the CW pump itself, hence spatial resolutions of centimeters have been demonstrated [34,35] with high strain or temperature resolution. Although pre-pumping has enhanced the acoustic wave, it induces a minimum Brillouin frequency shift for small section of temperature or strain change to be equivalent of at least 1/2 of a Brillouin spectral width, as the intensity of the non-changed stress or temperature section is much larger than that of the changed stress or temperature section, at 1,550 nm it is 10 MHz which is equivalent to 200 micro-strains, unless a dark base is implemented [38], which removes the pre-pumping induced depletion effects through an inverse background of the pulse. This approach allows high frequency and spatial resolution over longer sensing lengths. However, the time duration of the dark base depends on the sensing fiber length and pulse width. This means one must check the dark base length for each different sensing length and spatial resolution. It makes the implementation of this technique in field difficult.

More recently, a differential pulse-width pair Brillouin optical time-domain analysis (DPP-BOTDA) for high spatial resolution sensing has been proposed, still using the pre-pumping idea to measure the differential Brillouin gain signal instead of the Brillouin gain itself [39,40]. This technique uses two different pulses of nearly identical duration, having high extinction ratios of 40–50 dB to remove the pre-DC effect. Two separate measurements are implemented with respect to the individual pulses, and the differential Brillouin gain signal is then obtained by subtraction of the two Brillouin signals [17,35]. The differential technique can be realized by two methods: π-phase-shift pulse pair [38] and differential pulse-width pair [39]. The idea of π-phase-shifted pulse added to non-phase-shifted pulse is similar as that of a bright pulse being added to a dark base [38], which is intensity modulation rather than phase modulation format.

In the π-phase-shifted pulse pair, two pulses share the same pulse-width except that the last portion of the second pulse is phase inverted (π phase shift). As a result, the spatial resolution is limited by the fall-time of the pump modulation and the phenomenon of secondary “echo” signals as was previously proposed. The differential pulse-width pair on the other hand, simply uses two pulses with different pulse-widths. Both techniques have shown the ability to achieve high spatial resolution and long-range sensing [40,41], however the π-phase-shifted pulse pair yields a 3-dB improvement in signal at the cost of a complicated data recovery process, due to the compound Brillouin spectrum created by two consecutive pulses of different phase.

The best performance of a differential Brillouin gain signal system has been a 2 km sensing length with 2 cm spatial resolution, for a Brillouin frequency shift resolution of 2 MHz which is equivalent to 2 °C temperature resolution [42]. For long sensing lengths, performance examples include a 30 cm spatial resolution for a 25 km sensing length having a Brillouin frequency shift accuracy of 1 MHz [17], and 0.3 MHz accuracy of 1 m spatial resolution over a 12 km sensing length [43]. While the best performance with the Brillouin echoes techniques (π phase shift) has been a 5 km sensing length with 5 cm spatial resolution, and 3 MHz Brillouin frequency shift [44].

Further exploration of DPP-BOTDA technology requires its realization in the optical field domain based on coherent interaction of the Brillouin gain and loss via optical differential parametric amplification (ODPA) [45]. Because it is not a Brillouin gain or loss as demonstrated in DPP-BOTDA, ODPA provides a combined Brillouin gain and loss process with two acoustic waves created by both carrier and Stokes, and carrier and anti-Stokes waves. Because the same carrier wave is involved in both the Stokes and anti-Stokes wave generation processes, the carrier wave “sees” the phase shifts from “gain” and “loss” processes, and both enhanced acoustic waves experience interference. As a result, a narrowed parametric Brillouin gain spectrum is generated.

## 8. Frequency Domain Sensor of Brillouin Optical Correlation-Domain Analysis (BOCDA)

The spatial resolution improvement in Sections 6 and 7 are based on time domain, but there is another technique based on frequency domain, BOCDA [46,47]. The spatial resolution of the BOCDA system is determined by the modulation parameters (amplitude and frequency) of a light source, rather than by the decay time of an acoustic wave. In this approach, two CW light waves with a Brillouin frequency difference are injected from both ends of the fiber. The effective Brillouin gain spectrum is built up upon a summation of the Brillouin signal at all positions of the sensing fiber. The signal processing of correlation between different positions provides a sharp peak for the matched Brillouin frequency, and the spatial resolution of BOCDA can be as high as 1 cm [47], even 3 mm spatial resolution has been demonstrated with dynamic measurement [48]. For these examples the sensing range has been limited to meters, which is imposed by the recovery of the relative phase shift between two CW waves.

With a double lock-in amplifier and a single-sideband (SSB) modulator to stabilize both probe and pump waves at different frequencies, the sensing range has been improved to 300 m. However the spatial resolution has been increased to 20 cm [49]. This technique introduces its own complexities, including a trade-off between spatial resolution and range.

## 9. Polarization Effect on the Brillouin Frequency

In the previous sections we have identified the best performance to date of distributed Brillouin sensors in terms of shortest spatial resolution and longest sensing length. In this section we will discuss the fundamental limit of the temperature and strain accuracies.

Brillouin frequency resolution is one of the key parameters that determine the sensor’s accuracy of temperature or strain measurement. Currently, it is widely believed that frequency resolution is ultimately determined by extracting the Brillouin peak frequency through fitting of the measured Lorentz spectrum. The minimum detectable change of the sensor can be denoted by the well known equation [50]:

(18) $${\delta \upsilon }_{\mathrm{B,SNR}}=\frac{1}{\sqrt{2}}\frac{\Delta \upsilon_{B}}{{\left(\mathrm{SNR}\right)}^{1/4}}$$

Here *SNR* is electric signal to noise ratio, Δ*ν_B_* is full width at half maximum (FWHM). For the pulsed BOTDA, Δ*ν_B_* represents the convolution of the pulse spectrum and natural Brillouin line width.

We know, however, the imperfections of the fiber core size and density non-uniformity in SMF-28 will cause the two possible polarizations to propagate at different phase velocities, dependent upon fiber location, and this is the so-called polarization mode dispersion (PMD) [51]. As a result, even if temperature and strain remain constant the Brillouin peak frequency changes at different fiber locations due to the varying birefringence (as it is related to *n_eff_*). This means that even if *SNR* can be very high, e.g., 60 dB, the uncertainty of the measured Brillouin frequency due to PMD will not improve. The introduction of a polarization scrambler has removed some of the polarization induced fluctuation on the Brillouin gain, however due to large PMD introduced by the scrambler itself on the Brillouin frequency measurement; it has reduced the strain and temperature sensitivity of the Brillouin frequency dependence contributed by the birefringence change, and the response time of the sensor system due to the speed of a scrambler.

The impact of PMD effect on the Brillouin scattering process has been previously studied in detail from the aspect of Brillouin gain variation [8,52]. It is demonstrated both by theory and experiment that the maximum Brillouin gain occurs when pump and probe waves have identical polarization, and the maximum gain is twice the minimum Brillouin gain. Recently, it has been found that the output state of polarization (SOP) of an arbitrarily polarized input probe wave would converge to an output SOP corresponding with the maximum Brillouin gain, through a so-called polarization pulling force at the high Brillouin gain condition [53]. This pulling effect as well as the local birefringence of SMF altogether govern the SOP evolution of pump and probe waves. In fact, PMD not only affects the SOP of the output probe wave, but also the peak Brillouin frequency, because *δn_induced_*(*z*) = *γ*|*E⃗_pump_*(*z*) + *E⃗_probe_*(*z*)|^2^ follows the changes in SOP of the pump and probe waves due to local changes in fiber polarization characteristics. This means the Brillouin frequency will also change along the fiber due to non-uniform density and non-circular shape variations of the fiber core. Obviously, the Brillouin frequency varies with the SOP of the pump and probe waves at different locations, and so do the Stokes and anti-Stokes frequencies, because it is unlikely that *n_eff_* (*ω*_0_ + Ω*_B_*, *z*) ≡ *n_eff_* (*ω*_0_ − Ω*_B_*, *z*), especially when we consider two polarization modes, when PMD exists in the fiber, because of the limited bandwidth of SOP. This means PMD introduces two error sources: (1) on the Brillouin frequency itself due to birefringence change at different location; and (2) on the Brillouin gain dependence from the local SOP change of the pump and probe wave. For the SNR of this distributed sensor system to be 60 dB with a Brillouin spectral width of 20 MHz, a Brillouin frequency uncertainty is of 0.44 MHz according to Equation (18).

This uncertainty is slightly different in spontaneous and stimulated Brillouin scattering due to the induced *Δn* change. The introduction of a PMD scrambler would itself introduce the peak frequency uncertainly of about 0.5–1 MHz depending on the speed of scrambler and average number of waveforms. However, SOP variations in the fiber create a range of the measured Brillouin peak frequency: on the order of 0.1 MHz for spontaneous Brillouin scattering *δν_B,SOP_*, and 0.4 MHz for SBS *δν_SBS,SOP_*, even though the Lorentzian shape fitting error of the spontaneous BS and SBS is 0.1 MHz in both cases. The ultimate temperature and strain resolution should be the combination of these two contributions:

(19) $${\delta \nu }_{B}(z)=\delta \nu_{\mathrm{B,SOP}}(z)+\delta \nu_{\mathrm{B,SNR}}(z)$$

## 10. Simultaneous Temperature and Strain Sensing Using the Distributed Brillouin Sensors

One problem with the implementation of Brillouin scattering based sensing systems in the field is the sensitivity of the Brillouin frequency shift to both strain and temperature. This effect leads to ambiguity in the measurement, as one does not know whether the observed frequency shift was caused by the change of strain or temperature. In a laboratory environment, the temperature is essentially constant and its effects can generally be neglected when measuring strain. In many field conditions this is not the case. An early solution to this problem proposed the use of two fibers placed adjacent to one another, in which one fiber was isolated from any strain effects [54]. The isolated fiber would be used to monitor the temperature, while the other fiber would measure the effect of both strain and temperature. Innovative approaches to measuring the strain and temperature simultaneously have been developed recently.

By combining the Landau-Placzek ratio with the frequency shift, the temperature and strain were determined simultaneously (in the spontaneous Brillouin scattering regime) at a spatial resolution of 40 m [55]. Using the same principle, an improved system has been reported, which achieved a temperature resolution of 4 °C, a strain resolution of 290 μɛ and a spatial resolution of 10 m for a sensing length of 15 km using LEAF fiber [56]. The analysis of both Brillouin gain (*i.e.*, intensity) and Brillouin shift provides better performance for simultaneous temperature and strain measurements over the standard communication fiber: it gives 3 m spatial resolution at a temperature resolution of 3 °C and strain resolution of 180 μɛ [57].

Centimeter resolution has been achieved with simultaneous temperature and strain sensing using Brillouin loss based distributed sensors based on polarization maintaining fibers (PMF) and photonic crystal fibers (PCF). The simultaneous temperature and strain sensing using the PMF can be realized with Stokes power (intensity), line-width and Brillouin frequency. Unlike the single mode fiber where the intensity fluctuation is overwhelmed by the PMD induced polarization changes, the intensity fluctuation in PMF is only caused by power fluctuations of the light source, provided temperature and strain coefficients are constant along the fiber and the light is launched in one of the PMF principal axes. If the intensity change of the light source is negligible, then intensity changes caused by temperature and strain can be measured accurately [58,59]. Furthermore, when frequency-stabilized lasers are used, the line-width of the laser is very narrow and changes slowly with time, allowing for accurate correlation of the Brillouin spectrum width with temperature and strain. Thus, three parameters (Brillouin frequency, Stokes power, and Brillouin spectral width) can be simultaneously used for temperature and strain measurements. Resulting in temperature and strain uncertainties have been listed for different pairs of these parameters in the following Table 1 [60]. This table provides performance examples for simultaneous temperature and strain measurements with three kinds of PMFs using Brillouin power, line-width, and peak frequency.

In PCFs with a solid silica core, the guiding mechanism is the same as in conventional SMF, except the effective cladding index is the average of the air and silica refractive indices. The solid silica core with a Ge-doped center region can increase the nonlinear refractive index of the core and create a smaller mode field diameter. As a result, the Brillouin spectrum of PCF shows multiple peaks with comparable intensities [61], with a main peak and several sub-resonance peaks due to guided acoustic modes. In one example, a PCF with a partially graded Ge-doped core was used, with the main resonance and a peak originating from a higher-order guided longitudinal acoustic mode being identified for simultaneous temperature and strain measurement using their Brillouin frequency shifts alone. It gave high temperature and strain measurement accuracy with spatial resolution of 20 cm [62].

## 11. TDM-Based BOTDA

Instead of the pulse and continuous wave combination used in conventional BOTDA, in this scheme two pulses, both a probe pulse and a pump pulse are used to perform the measurement. The spatial resolution is still defined by the probe pulse width, while the sensing length is determined by the pump pulse width. The delay between the probe pulse and the pump pulse can be changed to select a sensing section where the probe pulse interacts with the pump pulse. The measurement of the entire sensing fiber is realized by implementing the measurement for each sensing section through changing the delay between the two pulses, which is named as time-division multiplexing of different fiber sections. Because the interaction length is only determined by the pump pulse width instead of the entire fiber, the pump power can be increased to enhance the Brillouin interaction in the individual section and improve SNR without excessive amplification of the probe pulse.

**Figure 7.**
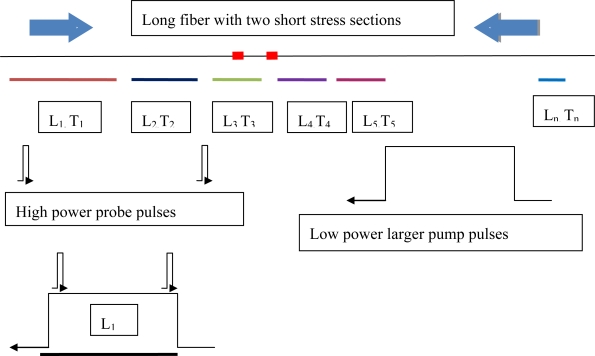
Time-division-multiplexing for 100 km sensing length.

The TDM-BOTDA is most effective on the same fiber type, for instance, SMF28. The effective sensing length is selected by a large pump pulse width as explained in Figure 7, and the differential Brillouin gain spectrum is measured with two smaller pulse widths of the probe waves, so that high spatial and temperature resolution can be obtained over the entire sensing length. The measurement of the entire sensing length is obtained by adding a sequence of the large pump pulses with each of them mapped by much smaller probe pulses; hence this is a time division multiplexing process. The key to this technique is to maintain equal gain over the entire fiber length.

While TDM-BOTDA allows long sensing lengths, the high spatial resolution is achieved with DPP-BOTDA [39]. In this way, high spatial resolution and long sensing length combined with high temperature resolution is possible. The combined TDM and differential BOTDA techniques offer higher precision than Raman amplification assisted BOTDA systems, because of “shorter effective length” and low Rayleigh scattering noise. In Raman assisted systems, not only does the Brillouin signal create Rayleigh noise, but also the Raman gain induced Rayleigh noise at the same wavelength as the Stokes frequency, which cannot be filtered. Hence the TDM-BOTDA with the combined differential Brillouin gain technique offers better performance than Raman assisted BOTDA for the same sensing length.

Using the TDM technique, we demonstrated a 100 km sensing fiber obtaining a spatial resolution of 0.6 m and 2 m at the end of 75 km and 100 km respectively, for a Brillouin frequency shift accuracy of 1.5 MHz, which is equivalent to 1.5 °C temperature resolution and strain resolution of 30 μɛ, respectively. Note, no inline optical amplifier is used within 100 km sensing length.

## 12. FDM-Based BOTDA

Another approach to increase the sensing length is to use non-uniform fibers, which were originally proposed for SBS slow light applications to reduce pulse distortion [63,64]. The idea is to have Brillouin amplification at different frequencies for distinct sensing fiber sections, so that the effective fiber length will be reduced to the resonant Brillouin frequency section, rather than the entire sensing length. Under this condition, the limitation of the sensing length is the fiber loss instead of the Brillouin gain saturation. The trade-off is the larger scan range, as different fiber sections represent different Brillouin frequency shifts. The choice of the fiber types and their order along the entire sensing length are critical in obtaining the highest SNR and the longest sensing length. We have used three types of fibers: MetroCor, LEAF and SMF-28, 25 km each for a 75 km total sensing length.

Table 2 gives the parameters of the three types of fibers used in the experiment.

**Figure 8.**
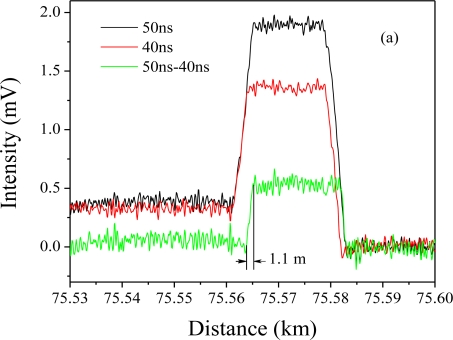
Time traces of the heated 18 m fiber using FDM-BOTDA with differential Brillouin gain technique. [26] (permitted by Nova Scientific Pub).

Figure 8 shows the measured strain at the end of the 75 km fiber using the setup as demonstrated in [26], where a segment of 18 m fiber is heated in an oven. The measurement is conducted with two pulses of 50 ns and 40 ns BOTDA, and through subtraction of two waveforms at similar pulse width we can get a spatial resolution of 1.1 m. The Brillouin spectrum fitting gives an uncertainty of the Brillouin frequency shift of 1 MHz, which is 1 °C temperature resolution and 17 μɛ strain resolution. This is the longest high performance distributed Brillouin sensor demonstration having a single pulse without inline amplifiers. Thanks to the DPP-BOTDA technique [39], the DPP of the FDM-BOTDA system gives the highest performance for 75 km sensing length. To increase the sensing length and maintain 1 m spatial resolution and 1 MHz Brillouin frequency shift, an inline EDFA has been added to double the sensing range to 150 km. At this doubled sensing length, we have recently achieved 2 m spatial resolution with 1 MHz Brillouin frequency shift uncertainty.

The performance of FDM-BOTDA is even higher than TDM-BOTDA, because different Brillouin frequencies in different fiber sections have reduced Brillouin interaction length, and hence lower the impact of SPM. The Brillouin gain of the pump signal at different sections cannot be added as in TDM-BOTDA, where the fiber is the same along the entire sensing length.

## 13. Combining Brillouin Gain and Loss

**Figure 9.**
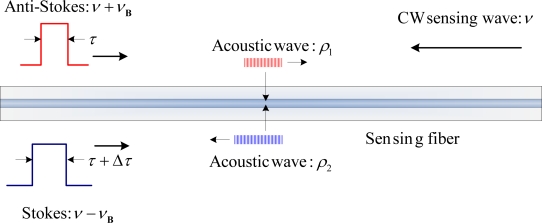
Schematic diagram of the ODPA-BOTDA [45] (Copyright © 2010 OSA, Reprinted with permission).

DPP-BOTDA offers a significant improvement in spatial resolution at the expense of doubled measurement time. In this section we explore a different approach to make single measurement and achieve differential Brillouin gain, based on coherent interaction of the Brillouin gain and loss via optical differential parametric amplification (ODPA). Coherent interaction of the Brillouin gain and loss can be applied to reduce the pump depletion effect in the distributed Brillouin sensor based on frequency domain analysis [39]. In our proposal, instead of using two continuous waves to perform the Brillouin gain and loss process, two long pulses with a small pulse width difference at the Stokes and anti-Stokes frequencies, interact with the CW sensing wave through two counter-propagating acoustic waves, creating Brillouin gain and loss at the same position simultaneously. At the overlapping region of the two long pulses, the gain and loss can eliminate each other when they are well balanced, which is “similar” to the subtraction process of DPP-BOTDA in the electrical domain. However, here it is realized in the coherent optical field domain via ODPA. This method has the same advantages as DPP-BOTDA, while the measurement time and noise are reduced by half due to one measurement instead of two, in addition unlike DPP-BOTDA, which uses subtraction of two different pulses of BOTDA, the ODPA technique carries the field subtraction in each fiber location directly. Thus, it is easier to prevent the photo-detector from becoming saturated by a large signal when the fiber length is long. On the other hand, in the ODPA-BOTDA sensor the signals have been cancelled in the optical field before reaching the photo-detector, and the ODPA-BOTDA sensor detects the signal difference directly. Therefore, the photo-detector in the ODPA-BOTDA sensor is less likely to become saturated, and even longer sensing length is possible. Because of the large pulse applied for both Brillouin gain and loss processes, this ODPA process maintains low saturation of the gain and low depletion of the loss, hence the sensing length can be longer than that in the normal BOTDA sensor. Figure 9 shows the scheme of ODPA-BOTDA, and the process can be described by Equation (20) via the slowly varying amplitude approximation and by adopting the same notations and symbols as in reference [14].

(20) $$\{\begin{array}{l} -\frac{\partial A_{1}}{\partial z}+\frac{n}{c}\frac{\partial A_{1}}{\partial t}=\frac{i\omega_{1}\gamma_{e}}{2nc\rho_{0}}{\tilde{\rho }}_{1}A_{2}+\frac{i\omega_{1}\gamma_{e}}{2nc\rho_{0}}{\tilde{\rho }}_{2}^{*}A_{3}-\frac{1}{2}\alpha A_{1}\\ \frac{\partial A_{2}}{\partial z}+\frac{n}{c}\frac{\partial A_{2}}{\partial t}=\frac{i\omega_{2}\gamma_{e}}{2nc\rho_{0}}{\tilde{\rho }}_{1}^{*}A_{1}-\frac{1}{2}\alpha A_{2}\\ \frac{\partial A_{3}}{\partial z}+\frac{n}{c}\frac{\partial A_{3}}{\partial t}=\frac{i\omega_{3}\gamma_{e}}{2nc\rho_{0}}{\tilde{\rho }}_{2}A_{1}-\frac{1}{2}\alpha A_{3}\\ (-2i\Omega_{1}+\Gamma_{B})\frac{\partial {\tilde{\rho }}_{1}}{\partial t}+(\Omega_{B}^{2}-\Omega_{1}^{2}-i\Omega_{1}\Gamma_{B}){\tilde{\rho }}_{1}=\frac{\gamma_{e}q_{1}^{2}}{4\pi }A_{1}A_{2}^{*}\\ (-2i\Omega_{2}+\Gamma_{B})\frac{\partial {\tilde{\rho }}_{2}}{\partial t}+(\Omega_{B}^{2}-\Omega_{2}^{2}-i\Omega_{2}\Gamma_{B}){\tilde{\rho }}_{2}=\frac{\gamma_{e}q_{2}^{2}}{4\pi }A_{3}A_{1}^{*} \end{array}$$

**Figure 10.**
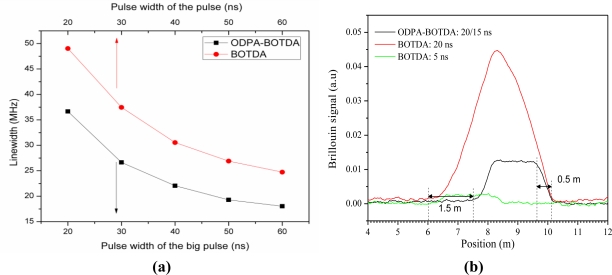
Brillouin spectrum width of the BOTDA (20–60 ns pulse) and the ODPA-BOTDA sensor with 20/18 to 60/58n s pulse pair based on Equation (20) (a, left); time domain signal of ODPA-BOTDA *vs.* BOTDA (b, right) [45] (Copyright © 2010 OSA, Reprinted with permission).

Here, *α* is the fiber attenuation coefficient, and Γ*_B_* is the inverse phonon lifetime. In the parametric amplification process, the interaction between optical fields *E*_1_, *E*_2_, and acoustic field *ρ̃*_1_ is a Brillouin loss process. *E*_1_ also interacts with *E*_3_ via acoustic field *ρ̃*_2_, which is a Brillouin gain process. The effect of Brillouin loss and Brillouin gain on optical field *E*_1_ is a coherent process of field addition rather than intensity addition. From Equation (20) it is clear that in the equation for *A*_1_, *E*_1_ is associated with *E*_2_ via *A*_2_, and *E*_3_ via *A*_3_, simultaneously. Due to the well balanced energy transfer between the Brillouin gain and loss processes, the Brillouin spectral width will not be the same as that of the gain or loss processes alone as shown in Figure 10a. Apparently, for different pulse widths, the line-width of the Brillouin spectrum in the ODPA-BOTDA sensor is always narrower than the line-width of the Brillouin spectrum in the normal BOTDA sensor, which is a result of the coherent differential parametric amplification process.

Figure 10b shows the experimental result of using combined Brillouin gain and loss processes for two pulses of Stokes and anti-Stokes waves having different widths of 20/15 ns. The 2 m strain section is measured with the ODPA-BOTDA technique shifted by 1.5 m, corresponding to the 15 ns pulse of the 20/15 ns pulse pair. This in turn reflects the time during which the signal increases from 10% to 90% of its peak value, and results in a spatial resolution of around 0.5 m, much smaller than the 2 m spatial resolution of the BOTDA sensor with 20 ns probe pulse that is measured in the same way as the ODPA-BOTDA sensor. The spatial resolution of the BOTDA sensor with a 5 ns probe pulse is comparable to that of the ODPA-BOTDA sensor with a 20/15 ns pulse pair; however, the signal amplitude of the ODPA-BOTDA sensor with a 20/15 ns pulse pair is 5 times greater than that of the BOTDA sensor with a 5 ns pulse. Because the Brillouin gain increases very slowly during the acoustic wave generation process, the 5 ns pulse in the BOTDA sensor can only get very small direct Brillouin gain. However, for the 20/15 ns pulse pair with much longer interaction time than the phonon lifetime (∼10 ns) in optical fiber, two acoustic waves can build up fully, so a larger differential gain is expected.

## 14. The Brillouin Grating as a Point Sensor

In this section we present another example of the Brillouin parametric amplification by writing a Brillouin grating in optical fiber with a length determined by two pump pulses [65]. The Brillouin grating can be used for measuring temperature via birefringence [66], and for performing distributed birefringence measurement [67] via two identical optical pulses counter-propagating with the frequency difference of the Brillouin resonance in one of the principal axes of a polarization maintaining fiber. Their overlapping region forms a moving grating, which can be set at any position in the fiber determined by the relative time delay between two pulses. This moving “FBG”, in fact, is the time-varying change in the dielectric constant induced by beating two pump beams through the electrostriction effect, namely SBS. The size of the grating is determined by the pulse length. The lifetime of this Brillouin grating is limited by the phonon lifetime (10 ns). The grating spectrum is a convolution of the pulse and Brillouin gain spectra. Such a Brillouin grating can be probed with a 3rd pulse launched on the orthogonal axis; the optimum frequency of the 3rd pulse is related to the local birefringence between two axes of PMF. This means, the grating feature is affected by the temperature and strain reflected in the Brillouin frequency and the local birefringence from two axes of PMF, and this local Transient Brillouin Grating (TBG) [68–70] can be used for the purpose of sensing. When we change the relative delay between two pump pulses, distributed sensing can be realized for the entire fiber. Because the large frequency shift associated with birefringence change is on the order of 40–50 GHz, the measurement accuracy of temperature and strain can be much higher than direct Brillouin gain spectrum measurement. It has been reported that 0.08 °C and 3 μɛ can be achieved with a continuous wave (without location information) [68] and pulse of 2 ns, equivalent to 20 cm of spatial resolution, temperature and strain accuracy are 0.4 °C and 9 μɛ respectively [69]. For BFS measurement, the differential pulse-width pair Brillouin optical time-domain analysis (DPP-BOTDA) is used to realize a high spatial resolution, a high spatial resolution of 20 cm is achieved by using a pulse-width difference of 2 ns and a narrowband Brillouin gain spectrum is obtained from a pulse pair of 30/28 ns. For birefringence-induced frequency shift (BireFS) measurement, two short pump pulses (2 ns) are used to generate a local Brillouin grating and thus get a high spatial resolution of 20 cm. The temperature and strain range can be up to 700 °C and 14 mɛ due to the short pulse (2 ns) based TBG.

In terms of using the temperature and strain measurements as a point sensor, the TBG behaves similar to a fiber Bragg grating (FBG) sensors [70], except the FBG sensor is a permanent grating, while TBG has a lifetime of 10 ns with variable length and position determined by the writing process and it is a moving grating. The sound waves can be treated theoretically by considering the time-varying change Δ*ɛ̃* in the dielectric constant associated with the acoustic density variation Δ*ρ̃*. It is usually adequate to assume Δ*ɛ̃* scales linearly withΔ*ρ̃*, as:

(21) $$\Delta \tilde{ɛ}=\gamma_{e}\frac{\Delta \tilde{\rho }}{\rho_{0}}$$

where *ρ*_0_ denotes the mean density of the material (e.g., glass), and *γ_e_* is the electrostriction constant. Recalling that the material density distribution can be expressed as *ρ̃* = *ρ*_0_ + [Δ*ρ̃*(*z*,*t*)+c.c.], and taking Δ*ρ̃*(*z*,*t*) = Δ*ρe*^*i*(*qz*−Ω*t*)^ to be the steady-state expression, yields:

(22) $$\Delta \rho ={ɛ}_{0}\gamma_{e}q^{2}\frac{A_{1}A_{2}^{*}}{\Omega_{B}^{2}-\Omega^{2}-i\Omega \Gamma_{B}}$$

**Figure 11.**
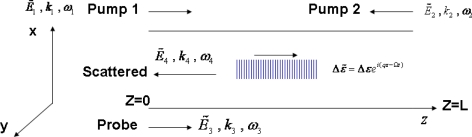
Brillouin grating generation and reading process.

Here *ɛ*_0_ is the vacuum permittivity; *q* ≈ 2*k*_1_ and *k*_1_ are the propagation constants of the acoustic wave and pump 1 (see Figure 11), respectively; Ω*_B_* and Ω are the angular Brillouin frequency and the frequency difference between the two pump beams, respectively; Γ*_B_* is the phonon decay rate which is inversely proportional to the phonon life time; *A*_1_ and *A*_2_ are the complex amplitude of the two pump beams, and * denotes the complex conjugate.

The interaction of the probe field *Ẽ*_3_ (*z*, *t*) = *A*_3_ (*z*, *t*) exp[*i*(*k*_3_*z* − *ω*_3_*t*)] + c.c. with the acoustic wave of propagation constant *q* produces a scattered wave *Ẽ*_4_ (*z*, *t*) = *A*_4_ (*z*, *t*) exp[*i*(−*k*_4_*z* − *ω*_4_*t*)] + c.c.

with frequency *ω*_4_ = *ω*_3_ − Ω. The interaction is assumed to be nearly phase-matched, e.g., *k*_4_ is very close to *q* − *k*_3_. The total electric field *Ẽ*(*z*, *t*) = *Ẽ*_3_ (*z*, *t*) + *Ẽ*_4_ (*z*, *t*) should satisfy the wave equation:

(23) $$\nabla^{2}\tilde{E}(z,t)-\frac{n_{y}^{2}+\Delta \tilde{ɛ}}{c^{2}}\frac{\partial^{2}\tilde{E}}{\partial t^{2}}=0$$

where Δ*ɛ̃* is given by Equations (21); *n_y_* is the refractive index of the *y* axis of the PMF without acoustic waves; and *c* is the velocity of light in vacuum. Since Δ*ɛ̃* oscillates at frequency Ω, it couples the optical waves at frequency *ω*_3_ and *ω*_4_ = *ω*_3_ − Ω.

By considering two portions of Equation (23) that oscillate at *ω*_3_ and *ω*_4_, separately, and introducing the slowly varying envelope approximation, we obtain (considering steady state limit):

(24a) $$\frac{{\partial A}_{3}}{\partial z}=\kappa_{1}A_{4}e^{-i\Delta kz}$$

(24b) $$\frac{{\partial A}_{4}}{\partial z}=\kappa_{2}A_{3}e^{i\Delta kz}$$

where Δ*k* = *k*_4_ + *k*_3_ − *q* is the phase mismatch, $\kappa_{1}=\frac{i\omega_{4}^{2}\Delta ɛ}{2k_{3}c^{2}}$ and $\kappa_{2}=-\frac{i\omega_{3}^{2}\Delta {ɛ}^{*}}{2k_{4}c^{2}}$. The general solution for Equations 24(a,b) are as follows:

$$\begin{array}{l} A_{3}(z)=(C_{1}e^{\mathrm{gz}/2}+C_{2}e^{-gz/2})e^{-i\Delta kz/2}\\ A_{4}(z)=(D_{1}e^{\mathrm{gz}/2}+D_{2}e^{-gz/2})e^{i\Delta kz/2} \end{array}$$

with *g*^2^ = 4*κ*_1_*κ*_2_ − (Δ*k*)^2^, where *C*_1_, *C*_2_, *D*_1_ and *D*_2_ could be determined by the boundary condition. If we consider the boundary condition to be:

$$\{\begin{array}{c} z=0:A_{3}=A_{3}(0)\\ z=L:A_{4}=0 \end{array}$$

we obtain:

(25a) $$A_{3}(z)=A_{3}(0)\frac{g\;\text{cosh}[\frac{g}{2}(z-L)]+i\Delta k\;\text{sinh}[\frac{g}{2}(z-L)]}{g\;\text{cosh}(\frac{g}{2}L)-i\Delta k\;\text{sinh}(\frac{g}{2}L)}e^{-\frac{i\Delta kz}{2}}$$

(25b) $$A_{4}(z)=A_{3}(0)\frac{2\kappa_{2}\text{sinh}[\frac{g}{2}(z-L)]}{g\text{cosh}(\frac{g}{2}L)-i\Delta k\text{sinh}(\frac{g}{2}L)}e^{\frac{i\Delta kz}{2}}$$

**Figure 12.**
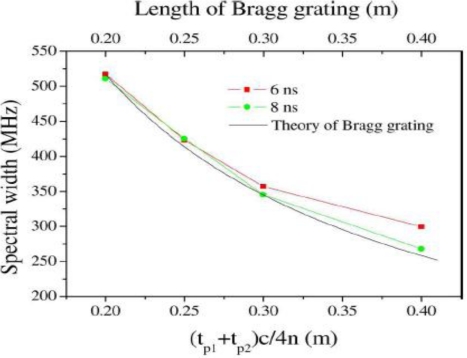
The intrinsic Brillouin grating spectral width as a function of length [71] (Copyright © 2010 OSA, Reprinted with permission).

Equation (25) is similar to those used for modeling FBGs [70], except the gain g carries the power of two pump signals. For weak gratings (small g), the bandwidth of the Brillouin grating is independent of Brillouin scattering line-width, $\Delta \upsilon \;=\frac{c}{2\mathrm{nL}}$, yielding the same relation used for Bragg gratings. Figure 11 shows the Brillouin grating and its formation by two pump waves with the frequency difference of the Brillouin frequency shift. The spectral width of the Brillouin grating is illustrated in Figure 12. In the x-axis, the two pump pulses for creating gratings are t_p1_ and t_P2._

The results in Figure 12 prove directly that the moving Brillouin grating and the static fiber Bragg grating follow the same basic theory. The other important characteristic of the Brillouin grating is the acoustic wave relaxation or grating decay. The Brillouin grating is created through the electrostriction effect and can only be sustained by maintaining the two pump waves. After these waves are removed, the Brillouin grating will exponentially decay, which characterizes an intrinsic Lorentzian Brillouin gain spectrum. The smaller bandwidth Brillouin grating has been observed experimentally [72]. However, if the pulse width of the pump wave is smaller than the phonon lifetime, the grating spectrum takes on a Gaussian shape, which reflects the apodization of the Brillouin grating feature [71].

## 15. Dynamic Measurement Capability with the Brillouin Scattering

Dynamic strain response measurements are essential for structural fatigue evaluation due to seismic or man-made activities and material aging. So far, the distributed Brillouin sensor has been limited to static strain measurement. Early demonstration of distributed dynamic measurement was based on the BOCDA technique using CW light for pump and probe waves [73]. For one pre-determined location, dynamic strain applied at up to 8.8 Hz has been monitored for a 5 cm strained section.

In time domain measurement, one can use polarization dependence of the Brillouin gain to measure the vibration [74,75] or a dynamic heating process [76] without the need for sweeping the Brillouin frequency of the spectrum. Laboratory testing has demonstrated distributed measurement of a 13 Hz vibration frequency with 2 m spatial resolution over a 100 m sensing fiber, while for point sensor detection, a maximum vibration frequency of 10 kHz was possible. Such a vibration sensor has been used in the field [75]. The long measurement time of traditional distributed Brillouin sensors is avoided by eliminating the frequency sweep of the pump and probe lasers and instead locking them at a single beat frequency, corresponding to the static strain of the structure in which the fiber is embedded. This unique sensor allows distributed measurement of vibration frequencies along a sensing fiber of 300 m with a spatial resolution of 2 m.

## 16. Structural Health Monitoring with Distributed Sensor Based on Brillouin Scattering

Distributed Brillouin sensors are one of the most promising diagnostic tools for improving the structural health monitoring process, with the first such structural strain monitoring system reported in 1999 [77]. Structural Health Monitoring (SHM) has been used to identify early signs of potential problems in civil structures, to prevent disasters, and conduct needed repairs at the appropriate time, avoiding unnecessary costs and reducing economic burden. Thus, it is important to have accurate and real time monitoring on the safety assessment of civil structures, such as bridges, dams and pipelines. Currently such evaluations are carried out by engineers trained in visual inspection, which sometimes can be inaccurate due to differences in their personal experience with safety condition assessment. To increase the inspection efficiency and accuracy, fiber optic sensors are one of the most promising candidates, due to their features of durability, stability, small size and insensitivity to external perturbations, which makes them ideal for the long-term health assessment of civil structures. Optical fibers can cover the large areas of civil structures, safely access and monitor the status of these structures. This kind of sensor has the advantage of a long sensing range and the capability of providing strain or temperature at every spatial resolution along the entire sensing fiber, imbedded in or attached to the structures, using the fiber itself as the sensing medium [78,81].

Recent studies have been conducted to monitor structural strain of composite and concrete beams under limited load, *i.e.*, the structure responds to the load linearly with the average strain over the spatial resolution being monitored [78]. The extraction of average strain used single peak fitting or centroid method (integrate the spectrum area method) [78,80,82]. Those methods provide fast signal processing to identify critical strain points, while multiple peak detection [81] provides better strain accuracy at the cost of longer processing times, making it less suitable for dynamic detection and disaster prevention. If dynamic strain or temperature monitoring is required, intensity detection with fixed Brillouin scattering could be used without scanning the Brillouin spectrum [76], which reduces the measurement time significantly. The first few civil structural applications were tested under elastic working conditions in order to validate the suitability of the distributed Brillouin sensor for strain monitoring. In those cases no plastic deformation happened during the loading process. Most of the recent research of the distributed sensor has been focused on using Brillouin scattering as a tool to correlate measured strains with the civil structural condition. For example cracks and deformation, including the prediction of cracks in concrete structures and buckling in pipelines has been investigated using special signal processing schemes developed from detailed studies of the Brillouin spectrum shape change, with particular attention to asymmetry and broadening in addition to the peak change [83]. Using three factors instead one, the location of the deformation has been successfully identified using broadening factor [84].

For field demonstrations, the choice of the glue for embedding the fiber is critical, and depends on the material parameters of the structures. The choice of glue affects the strain transferring matrix. Normally harder glue applied in thinner layers is good for static strain measurement. For dynamic measurement, relatively soft epoxy glue is preferred as it is not easy to break fibers with repeatable bending and stretching, especially for the large extension measurement of a steel pipe. However for concrete and plastic pipes, due to small thermal extension, hardened glue gives better strain transferring ability.

A strain calibration pre-test is needed to verify the interface between structures and fibers works well, which means the strain can be transferred linearly. The signal processing approach is critical to monitor these changes in various structures subjected to heavy loads, or environmental conditions. The measured strain distribution of both the concrete columns and pipes provided detailed information on the structure’s health at the local and global level, associated with deformations, cracks and buckling. Such critical information is crucial to field engineers for making decisions pertaining to repairs and maintenance in order to protect public safety.

## 17. Conclusions

Fiber sensors based on Brillouin scattering have proven to be a powerful tool for distributed measurements of strain and temperature, and they have found a number of applications in practice as outlined earlier in this paper. 100 km sensing range is made possible without need of inline amplifiers inside the sensing length, for longer distances (>100 km), EDFAs have been added to improve FDM based BOTDA, achieving spatial resolutions of 2 m and temperature resolutions of 1 °C. The best spatial resolution is only limited by the resolution of the digitizer. For the state of the art technology; the time resolution is 25 ps, which is equivalent to 2.5 mm spatial resolution. The trade off is the low strain or temperature resolution and shorter sensing length, unless the recently proposed TDM and FDM based BOTDA are employed. Because of stress induced birefringence change on the Brillouin gain, one would expect dynamic strain measurement can be achieved at higher speed and better spatial resolution.

Although the performance of distributed sensors based on Brillouin scattering has improved significantly over the years, especially through pre-pumping of the acoustic wave via differential Brillouin gain technique the spatial resolution could be improved to 2 cm with a 2 km sensing length. The reduction of the effective Brillouin interaction length allowed achieving high precision distributed sensor over long sensing lengths (>150 km).

The main challenges remain as follows:

1. The polarization mode dispersion has introduced the Brillouin gain fluctuation and it also induced the uncertainty in Brillouin peak frequency measurement. Te introduction of a polarization scrambler has removed the polarization dependence of the Brillouin gain at the cost of (a) eliminating the dynamic measurement option, and (b) the uncertainty of the Brillouin peak frequency measurement of at least 1 MHz. Further increasing the temperature and strain accuracy requires new technique.
2. The simultaneous measurement of temperature and strain in standard single mode fiber at centimeter spatial resolution.
3. The dynamic measurement with high speed and centimeter spatial resolution over long sensing length.
4. To make the distributed sensor system cost effective, new measurement parameters should be explored, especially the option of the chemical or bio-related monitoring can be extremely attractive.

## Figures and Tables

**Table 1.** Uncertainty of temperature and strain calculated with measured Brillouin frequency (F), power (P), and bandwidth (B) from [60] (Copyright © 2004 OSA, Reprinted with permission).

Property	PANDA			Bow Tie			Tiger
	F–P	F–B	P–B	F–P	F–B	P–B	F–P
Uncertainty of temperature (°C)	8	2	38	4	3	38	16
Uncertainty of strain (*μɛ*)	153	39	135	237	126	195	598
Maximum error of ΔT (°C)	10	4	58	12	7	137	78
Maximum error of Δ*ɛ* (*μɛ*)	331	82	249	741	490	1096	1308
rms (ΔT) (°C)	2	2	40	8	4	73	38
rms (Δ*ɛ*) (*μɛ*)	221	43	149	414	152	567	926

**Table 2.** Parameters of three types of fibers.

Fiber type	Length (m)	*g_B_*/*A_eff_* (W^−1^m^−1^) ^a^	BFS (MHz)	Dispersion (ps/(nm·km)) ^b^
MetroCor	25	0.1669	10518	−10 ∼ −1
LEAF	25	0.0892	10645	2 ∼ 6
SMF-28	25	0.1281	10867	16 ∼ 19

a These are average gains with scrambling the polarization state of the probe wave.

b The values are from fiber datasheets.
